# Changing Tides in the Treatment of Spondylodiscitis? A Retrospective, Monocentric Comparison of Mortality and Quality of Life After Surgical and Conservative Treatment

**DOI:** 10.3390/clinpract15110198

**Published:** 2025-10-29

**Authors:** Victoria Buschmann, Erik Wegner, Daniel Wagner, Alexander N. Wartensleben, Philipp Drees, Stefan Mattyasovszky, Tobias Nowak

**Affiliations:** 1 Department of Orthopaedics and Traumatology, University Medical Center of the Johannes Gutenberg University, 55131 Mainz, Germany; 2 Service D’orthopédie et de Traumatology, Centre Hospitalier Universitaire Vaudois, 1005 Lausanne, Switzerland

**Keywords:** spondylodiscitis, internal stabilization, antibiotic treatment, quality of life, mortality

## Abstract

**Background:** The increasing incidence of spondylodiscitis and its potentially severe consequences when not promptly diagnosed highlight the need for further research to improve treatment guidelines, reduce mortality and morbidity and improve the quality of life in patients who suffer from persistent physical limitations. **Methods:** We collected data from 103 patients, with 8 patients lost to follow-up, who were diagnosed with vertebral osteomyelitis, disk infection or discitis between 2009 and 2018. The primary outcome was the 1-year mortality rate in patients treated with either conservative or surgical intervention, with both groups receiving antibiotic treatment. A standardized questionnaire was used to assess health-related quality of life after treatment by evaluating the European Quality of Life 5 Dimension 5 Level version (EQ-5D-5L) and the European Quality of Life Visual Analog Scale (EQ-VAS). In addition, we used the Oswestry Disability Index (ODI) and the Parker Mobility Score to identify backpain-related limitations after treatment. **Results:** The group receiving surgical treatment had a significantly lower 1-year mortality rate (22%) than did those who were treated conservatively, who had a 4-fold greater risk for death after a year following first diagnosis of SD and treatment. With respect to quality of life, 39 patients answered the standardized questionnaires during follow-up, and the questionnaire results revealed no significant difference in limitations in daily life or in health-related quality of life, with a median Parker Mobility Score of 9 for the conservatively treated patients compared with 7.5 for the surgically treated patients. This difference between the groups was not statistically significant, with a *p* value of 0.216 > α. A similar result was obtained in the evaluation of the ODI, with a medial index of 30% in the conservatively treated group compared with 24% in the surgical group, which was not statistically significant as indicated by a *p* value of 0.360 > α. **Conclusions:** The early surgical approach when treating spondylodiscitis is advantageous for identifying the underlying infection and initiating appropriate antibiotic therapy, therefore reducing mortality and resulting in a greater likelihood of full recovery than the conservative treatment does.

## 1. Introduction

The incidence of pyogenic spondylodiscitis (SD) is increasing due to the increasing population of multimorbid and elderly people in the western world. It is the third most common form of osteomyelitis and affects the intervertebral disk and its adjacent vertebral bodies [1]. Depending on the severity of SD, the paravertebral soft tissue and the structure of the spinal canal may also be affected [2]. The close relationship between musculoskeletal and neuronal structures explains the heterogeneous clinical presentation of this potentially disabling condition, with a mortality rate of up to 32% [3]. Metaphorically speaking, SD is a chameleon, as the condition may be unapparent for a long time or manifest as nonspecific back pain. However, it can also be associated with neurological deficits or even fulminant sepsis. This heterogeneity in disease presentation is not only a challenge in terms of diagnosis but also leads to controversies regarding the most effective therapeutic strategies for its management [4].

The current treatment for SD includes surgical and conservative treatment options, namely, monotherapy with antibiotics with extension to surgical debridement versus internal stabilization followed by antibiotic medication. Ideally, antibiotics that target the pathogen are used in both treatment strategies [5].

The lack of evidence-based data, especially from prospective randomized controlled studies regarding SD therapy, has led to a lack of standardized decision-making for both surgical and conservative treatment. For a strict conservative approach, an expert consensus from a low level of evidence was established to optimize the duration and type of antibiotic treatment for the most likely or confirmed pathogen infection. The surgical approach is also based on an expert consensus, which gives the indications for a surgical approach in terms of age, comorbidities and the presence of spinal abscesses and neurological deficits [5].

Owing to the absence of prospective studies, it is necessary to use retrospective studies, and because of the lack of validity in retrospective studies, the data from these studies must be tested repeatedly.

The increasing incidence of SD and potentially substantial impairments in performing daily activities when it is not treated sufficiently show the importance of therapeutic guidelines for reducing mortality and morbidity and preserving the independence of each patient in everyday life. Although treatment guidelines for spondylodiscitis such as the 2015 Guidelines exist, further assessment of mortality and quality-of-life outcomes is warranted to better understand the benefits of surgical versus conservative management [5].

The aim of this study was to compare the two treatment protocols for SD and to determine which treatment group had better outcomes. The primary endpoints were 1-year mortality and 1-year overall survival. The surrogate endpoint considered quality of life with respect to patient-specific risk factors.

## 2. Materials and Methods

### 2.1. Study Design and Patient Recruitment

For this retrospective, monocentric cohort study, patient data from 2009 to 2018 at the Department of Orthopedics and Traumatology at the Mainz University Medical Centre, a tertiary care hospital in Germany, were analyzed. The data included only patients who underwent treatment with the following recorded diagnoses as defined in the german medical coding system which base on ICD- and OPS Codes: vertebral osteomyelitis, disk infection or discitis. All three of these diagnoses were established based on an integrated assessment of clinical presentation, laboratory parameters an imaging finding, supplemented by microbiological or histopathological confirmation when available. To collect the data, the clinical information system SAP i.s.h. med (SAP ERP 6.0 Release 2005) was used, and the information was pseudonymized. Patients with nonpyogenic infections of the spine were excluded. These included patients who tested positive for either *Mycobacterium tuberculosis* or a fungal infection.

This study was conducted in accordance with the tenets of the Declaration of Helsinki and approved by the Ethics Review Board of Rhineland-Palatine, Germany (2018-13714-retrospective).

### 2.2. Data Collection

The recorded data on each patient’s death were used to compare the 1-year mortality rates of patients treated with either surgical or conservative treatments. When no data were available, a survey was used to find any deaths that were not digitally documented.

With respect to the surrogate analysis questions, the cohort was tracked by mail to identify any persisting restrictions in daily activities and to gather information about the current health-related quality of life. For this purpose, standardized questionnaires were used, namely, the European Quality of Life 5 Dimension 5 Level version (EQ-5D-5L) and the European Quality of Life Visual Analog Scale (EQ-VAS). In addition, we used the Oswestry Disability Index (ODI) and the Parker Mobility Score (PMS) to identify backpain-related limitations after treatment.

**Figure 1:**
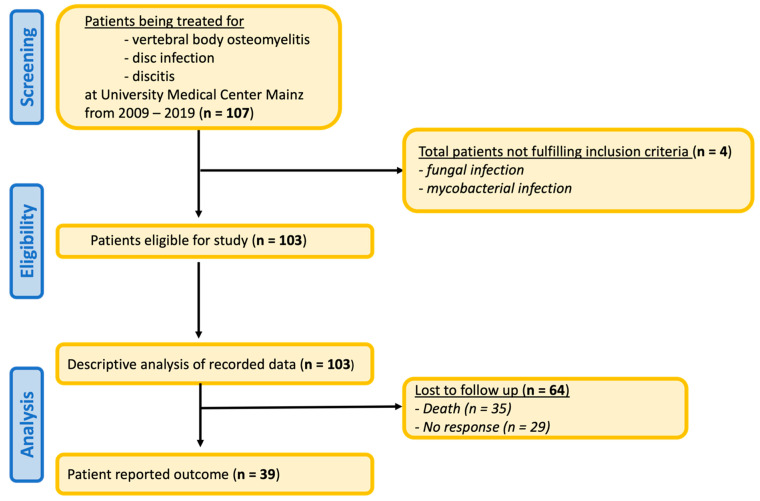
Patient Recruitment and Follow-up.

With respect to the recorded and digitally saved data for descriptive analysis, 107 patients were screened, with 4 patients not fulfilling the inclusion criteria because of the exclusion of pathogen data. In the context of surrogate analysis, 35 patients died before contact was made. Twenty-nine questionnaires remained unanswered; thus, 39 patients from the initial cohort of 107 patients could be evaluated [Figure 1].

The statistical programs IBM SPSS 29 and Microsoft Excel (Microsoft Corporation 2021 version) were used.

Among the 107 patients with a diagnosis eligible for inclusion, 4 could not fulfill the inclusion criteria due to fungal or mycobacterial infection, and 64 were lost to follow-up. Thirty-nine of the remaining 103 patients submitted questionnaires concerning their current health status.

### 2.3. Treatment

Conservative treatment involved initial antibiotic therapy based on identification of the pathogen whenever possible via an antibiogram-guided medication. In addition, when available, the kind of bacteria detected in the respective samples was recorded. If this was not feasible due to the inability to obtain a sample from a specific infection site, calculated antibiotics were initiated.

Complementary external immobilization was performed with a brace. Owing to inconsistent patient and course-related risk factors such as age, comorbidities and the presence of neurological symptoms, the duration of antibiotic treatment varied from 6 to 12 weeks. Whenever progression occurred, the treatment was intensified, ultimately leading to secondary surgical intervention.

The primary surgical approach included radical surgical debridement of all affected spinal levels, with internal stabilization performed as needed in cases of instability using dorsal or ventral instrumentation. Surgical treatment was then accompanied by antibiotics after the collection of intraoperative tissue samples. Depending on the laboratory results, the medication was started empirically or via an antibiogram. The duration of antibiotic therapy, ranging from 6 to 12 weeks, depended on the patient’s age, preexisting conditions, and the pathogen and followed the national German recommendation for the treatment of SD.

The decision of whether surgical or strict conservative treatments should be initiated depended mostly on present neurological deficits, spinal abscesses or deformities with the presence or absence of spine instability. Due to the lack of prospective, randomized controlled studies, recommendations in the current literature were implemented based on expert agreement and therefore had a low level of evidence informing the recommendations [5].

In cases of culture-negative SD, the therapeutic approach was not standardized. Treatment decisions, including the choice and duration of antibiotic therapy as well as the indication for surgery, were made individually based on clinical presentation, imaging finding and multidisciplinary discussion.

## 3. Results

### 3.1. Clinical Characteristics of Patients with Spondylodiscitis

For this study, 103 patients met the inclusion criteria with a diagnosis of vertebral body osteomyelitis, disk infection or discitis. The entire treatment period, as documented, was used to perform a descriptive analysis, and whenever possible, it was complemented with the results of the patient’s reported status. This approach led to individual follow-up periods for each patient. Whenever histopathological verification of bacterial presence was possible, it was documented. Although this was not the primary focus of the study, the corresponding results are presented in Table 1.

The outcome of any disease is closely connected to a patient’s individual characteristics and risk factors. The study population was 61% men and 39% women. Most patients were older than 65 years, with an average age of 77.45 years.

The manifestation of spondylodiscitis is closely dependent on comorbidities such as diabetes, cardiovascular diseases, and oncological diseases, which favor infection by suppressing the immune system, reducing vascularization and promoting opportunistic infections. Therefore, the cohort was screened for individual risk factors with the following results:

While 35% presented with preexisting diabetes with end-organ damage, 29% suffered from chronic kidney injury. In addition to patients with highly progressed diseases, the cohort included patients with heterogeneous conditions that needed to be factored in to identify their impact on outcomes and complications. For this purpose, the Charlson Comorbidity Index (CCI), which identifies the preexisting 10-year survival rate of a patient in terms of age and comorbidities with a predefined scoring system, was used.

**Figure 2:**
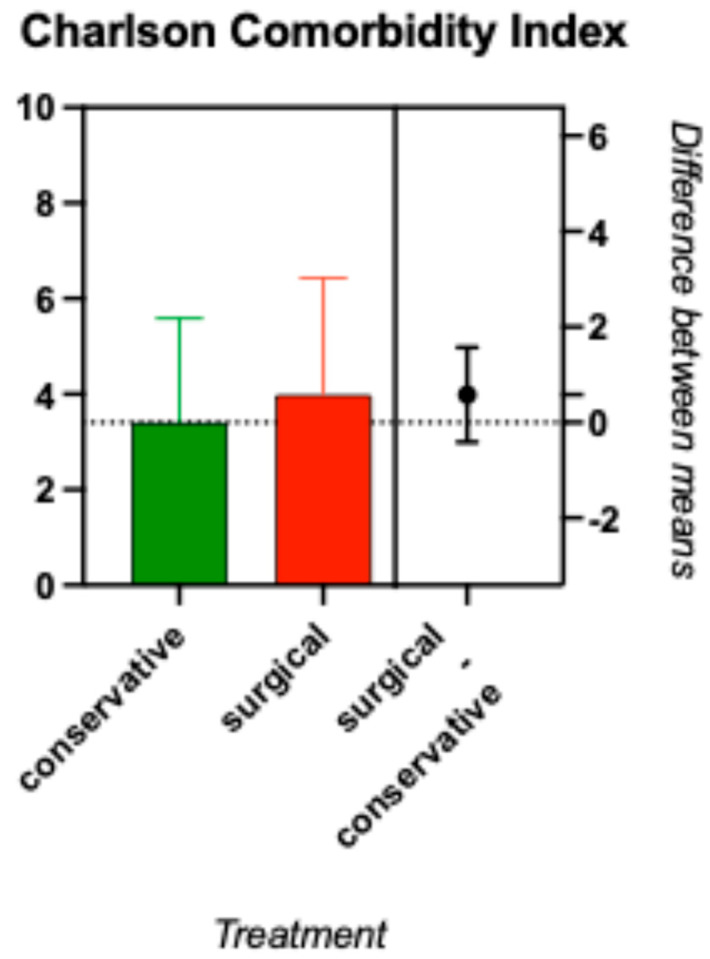
*t* test of the CCI comparing both treatment groups revealed no significant differences in the baseline comorbidities, with medians of 4 (conservative) and 5 (surgical).

In this study, the median CCI was 5 for nonsurgically treated patients and 4 for surgically treated patients, without a significant difference [Figure 2]. As a reference, the CCI-5 indicates a 1-year mortality of 85%, whereas 4 indicates a 52% risk for comorbidity-related death per year [6].

The results of the non-significant difference in CCI in both groups had impact on the following statistical testing, because we omitted the adjustment in the subsequent tests.

### 3.2. 1-Year Mortality

All deaths that contributed to the mortality rate had to result from SD or its treatment, i.e., those who died because of septic progression of the disease within a year following the first diagnosis of SD. If the cause of death was not attributable to the SD, e.g., because of a thromboembolic event, then those patients were excluded. Ultimately, 95 of the 103 patients were screened, while 8 were lost to follow-up in the first year after diagnosis.

**Figure 3:**
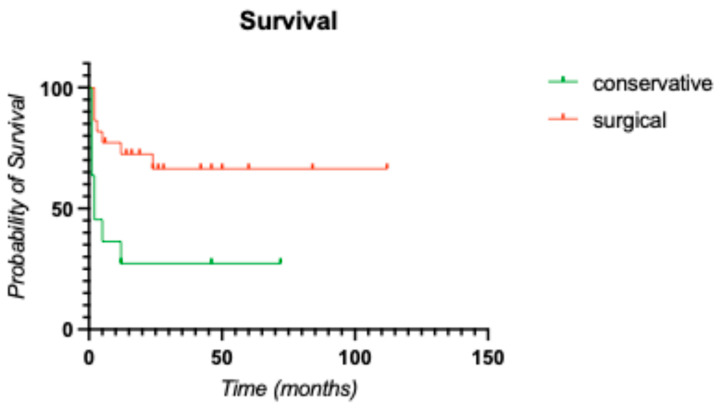
Kaplan-Meier survival curve comparing both groups. The overall mortality risk in surgically treated patients was 22%, whereas it was 40% in conservatively treated patients.

Among 95 patients, 32 received primarily conservative treatment, and 63 underwent additional surgery. Six of the patients from the former group died within one year after the initiation of treatment, whereas 3 of the latter groups died. The Kaplan-Meier curve revealed a higher mortality rate in the conservative study arm (40%) than in the surgical group (22%) after the end of the one-year observation period [Figure 3].

The statistical significance of this finding was confirmed via the log rank test, with a *p* value of 0.003 > α. Estimating the relative risk via the hazard ratio revealed a 4-fold greater risk of death within 1 year after conservative treatment than after surgical treatment, with an Exp(B) of 4.02.

### 3.3. Treatment Failure

In addition to 1-year mortality, therapy failure was also examined. Conservative treatment that resulted in patients with persisting signs of an infection or a recent onset of neurological symptoms or spinal abscesses was considered a failure in treatment, and a secondary surgical approach was initiated. Similarly, any revision surgery or reoccurrence of the infection in primarily surgically treated patients was documented as a failure in treatment.

Thirty-nine percent of the conservatively treated patients did not achieve a full recovery and had to undergo surgery, whereas the surgical approach led to a failure in treatment of only 19%. A chi-square test confirmed that this difference was statistically significant (*p* value of 0.032 < α).

The hazard ratio obtained from cox regression analysis is Exp(B) = 2.495, indicating a higher risk of failure when conservative treatment was used initially [Table 2].

### 3.4. Quality of Life

**Figure 4:**
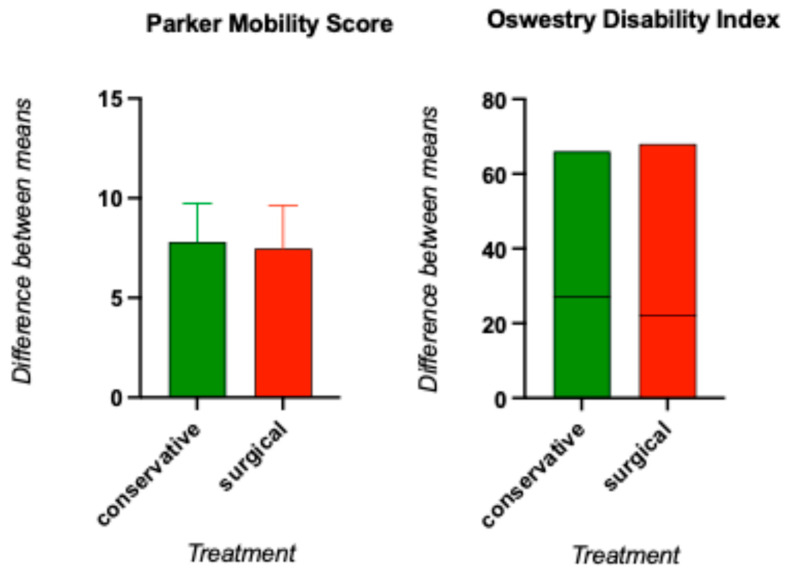
Comparison of the PMS rate and ODI between the conservative and operative treatment groups. Both parameters were not significantly different.

Some questionnaires sent to the 95 included patients were not completed and returned, mostly because of outdated addresses or because the patient was already deceased. We received 39 follow-up datasets that yielded the following results:

Regarding the questionnaire results concerning mobility as measured by the Parker Mobility Score, the median score of the conservatively treated patients was 9, whereas the surgically treated patients had a median score of 7.5. This difference between the groups was not statistically significant, with a *p* value of 0.216 > α given by the Mann-Whitney U test [Figure 4].

A similar result was observed in the evaluation of the ODI, with a median index of 30% in the conservatively treated group compared with 24% in the surgical group. The t test, however, with a *p* value of 0.360 > α, revealed that the treatment strategy had no significant effect on backpain-related limitations in daily activity. The patients in both groups therefore stated that they had moderate restrictions [Figure 4]. The results can be summarized as following: the higher the percentage, the greater the disability.

The same result was found when the EQ-5D-5L questionnaire results were examined. Both groups had a quality-of-life score of 0.828, indicating no describable difference between the two treatment options.

### 3.5. Adjusting for Sex and Comorbidities

Although the distribution of patient characteristics in the cohort reflected those in the total population of patients with SD, tests were used to confirm the effects of sex and multimorbidity on the results.

We corrected for the heterogeneity present in the surgically treated patient cohort, with a majority of these patients (66%) being male. Via the chi-square test, we determined that the treatment outcomes were independent from sex, with a *p* value_conservative_ of 0.790 and *p* value_surgical_ of 0.753, both being >α.

Most patients who entered the clinic had relevant comorbidities that could influence the outcome of either treatment. A Mann-Whitney U test was used to compare both groups, and a *p* value of 0.658 > α indicated that there was no significant difference between the groups. Additionally, with *p* values of 0.041 (conservative) and 0.047 (surgical) < α, the test revealed a significant influence of comorbidities on the outcome independent of the treatment.

## 4. Discussion

The primary aim of this study was to identify a treatment for SD that was more effective at reducing 1-year mortality and improving quality of life. Based on our data, we can conclude that a primary surgical approach reduces overall disease-related mortality, although this observation has to be put into perspective with the small cohort, loss to follow up and the broad confidence interval.

In terms of age and sex distribution, the cohort studied was comparable to the total population of patients with SD, with a median age of 69 years and approximately 1.5-fold more male patients, which is consistent with current epidemiology studies in the literature [6]. The choice of treatment had no significant effect on patient quality of life or mobility, in contrast to the negative effects of age on these parameters.

Surgery seemed to be a significantly more effective therapy, with 81% of patients in this treatment group achieving full recovery. It should nevertheless be acknowledged that full recovery, defined as the absence of revision or re-hospitalization, is inherently subjective and, given the substantial loss to follow-up, cannot be reliably assessed. It remains to be determined whether both treatments are equally feasible because early and aggressive surgery is recommended by experts for patients with preexisting neurological symptoms or abscesses.

Although the extent of the disease, which was determined by clinical, laboratory, and imaging findings, differed substantially, the baseline patient-related risk factors, namely, the comorbidities influencing patient physiological reserve, were evenly distributed. This suggests that, although this study primarily included patients with significantly progressed SD who underwent surgical treatment, patients with mild symptoms might benefit from early surgical treatment even before neurological symptoms occur. Patel et al. [7], who examined the treatment of patients with spinal abscesses, reported similar results, with 41% of their conservatively treated cohort requiring secondary surgery.

These data are consistent with the findings of a meta-analysis in 2023 by Thavarajasingam et al. [8], who reported an overall pooled failure rate of 21% in conservatively treated patients (n_studies_ = 21).

With respect to common recommendations for the treatment of SD, e.g., the 2015 practical guidelines outlined by the IDSA (The Infectious Diseases Society of America) that included a limited recommendation for early surgical interventions, delaying surgery is no longer advisable. The data from this study and the conclusion of the most recent meta-analysis both supports early radical debridement alongside antibiotic therapy [5].

A closer examination of the Parker Mobility Score, also known as the New Mobility Score, reveals it has only been validated in patients with hip fractures to assess mobility outcomes in daily life and overall mortality [9]. Although the Parker Mobility Score has high intertest reliability, whether it is suitable for patients with nontraumatic conditions, such as SD, is unknown.

Patients with SD suffer from a gradual increase in pain and mobility restrictions, whereas patients with hip fractures experience acute trauma with previously preserved mobility in daily life when patients with already reduced autonomy caused by dementia or neurological impairments are excluded. In hip fracture cases, most patients remain mobile even with reduced functional muscular reserve or capacity, in contrast to the chronic decrease in mobility in individuals with SD [10]. Therefore, it is questionable whether PMS can be used to compare the mobility loss caused by muscular atrophy with that caused by sarcopenia. Further studies need to evaluate whether the Parker Mobility Score/New Mobility Score can be used regardless of the underlying cause of limited mobility.

Thirty percent of the documented patients were diagnosed with reduced kidney function when preexisting comorbidities were considered. As shown by Lenz et al. [11] in 2022, a correlation between the GFR, postoperative mortality and length of stay may be assumed. The cohort included a diverse array of comorbidities so that predictive parameters might help with decision-making in favor of or against surgical intervention depending on preexisting conditions. To determine whether the endpoints of mortality and mobility in daily activities are significantly correlated in patients with SD, especially in relation to loss of kidney function, further testing is necessary to rule out any confounding variables.

Nevertheless, the overall limitation of this study is that it is retrospective in nature with only a small cohort and a high loss to follow up rate; therefore, the evidence in this study is not greater than level 3 [12]. The relatively high rate of loss to follow up represents a limitation. However, it also reflects the practical difficulties of achieving long-term follow up in patients with SD, given the chronic nature of the disease and the often complex comorbidity profile of the affected population.

Owing to ethical considerations, a randomized prospective study is not feasible in patients who are already showing neurological symptoms, which makes it necessary to rely on retrospective studies.

Another limitation of this study is the various follow-up periods, especially regarding the data on quality of life after treatment. Half of the cohort was lost to follow-up, but the follow-up period was different for each patient; therefore, it is not known whether some patients would have benefited from a longer recovery period or, conversely, would have shown a progression of the disease and continued to decline in later years.

Given that age is one of the key factors for reduced mobility, the results of the Parker Mobility Score need to be considered in light of the passing of time and, therefore, an aging cohort. The limitations in overall mobility cannot be attributed only to the form of treatment a patient received.

Notably, the duration of antibiotic therapy was different in the conservative group versus the surgical group. Therefore, the duration itself needs to be considered when looking at primary and secondary endpoints, especially since there are no clear recommendations for the overall length of antibiotic treatment, particularly with respect to intravenous or oral application. There is no consensus in the literature, and recommendations range from 10 days to 3 weeks of systemic therapy and up to 12 weeks of overall antibiotic therapy with oral medication [13,14].

Especially in culture negative SD the antibiotic treatment did not follow standardized guidelines and therefore leading to bias ranging from observer to confounding by indication as patients may have been treated with the antibiotic covering the broadest spectrum and therefore influencing the overall result.

In clinical practice, the delivery of intravenous antibiotics has drawbacks, as a peripheral intravenous line may not function properly, or administration may be delayed. Intermittent administration of antibiotics may result in suboptimal efficacy and therefore interfere with the evaluated endpoints.

Another limitation of the study is that the therapeutic approach in culture negative SD was not standardized. Treatment decisions were made on individual basis, which may have introduced variability in management and outcomes.

## 5. Conclusions

An early surgical approach showed some advantages for identifying the underlying infection and initiating appropriate antibiotic therapy. The data at hand shows a lower mortality rate and greater likelihood of full recovery than conservative treatment does, although the statistical significance has to be re-evaluated. No differences in quality of life were found between the two treatment arms, but further research is needed to identify whether patients without neurological symptoms or spinal abscesses benefit from early interventions likewise.

The high lost-to-follow-up rate, as a typical limitation of retrospective studies, underscores the need for further investigations, which should ideally be conducted as multicenter studies. A prospective study, although ethically challenging, could contribute to a better understanding of treatment outcomes.

## Figures and Tables

**Table 1:** Overview of Bacterial Pathogens by Treatment Group.

*Pathogen*	*Surgical*	*Conservative*
*Methicillin-Sensitive Staphylococcus aureus (MSSA)*	23	6
*Staphylococcus epidermidis*	14	0
*Enterococcus faecalis*	8	3
*Propionibacterium acnes*	8	1
*Escheria coli*	8	1
*Staphylococcus hominis*	3	1
*Methicillin-Resistant Staphylococcus aureus (MRSA)*	2	1
*Pseudomonas aeruginosa*	2	0
*Candida albicans*	3	0
*Mycobacterium tuberculosis*	1	1
*Mycobacterium avium*	1	0
*Serratia marcescens*	1	0
*Salmonella enteritidis*	1	0
*Streptococcus agalactiae*	1	1
*Streptococcus mitis*	1	0
*Streptococcus pyogenes*	1	0
*Streptococcus bovis*	0	1
*Streptococcus dysgalactiae*	0	2

**Table 2:** Hazard Ratio Treatment Failure.

Variable	B	SE	Significance	Exp(B)	95% CI Lower	95% CI Upper
Treatment Failure (Conservative vs. Surgical)	0.914	0.386	0.018	2.495	1.171	5.318

The relative risk for treatment failure was 2.495 times greater in conservatively treated patients than in the surgically treated group.

## Data Availability

No new data were created or analyzed in this study.

## Abbreviations

CCI	Charlson Comorbidity Index
EQ-5D-5L	European Quality of Life 5 Dimension 5 Level Version
EQ-VAS	European Quality of Life Visual Analog Scale
ODI	Oswestry Disability Index
PMS	Parker Mobility Score
SD	Spondylodiscitis
